# Climate Change-Induced Range Expansion of a Subterranean Rodent: Implications for Rangeland Management in Qinghai-Tibetan Plateau

**DOI:** 10.1371/journal.pone.0138969

**Published:** 2015-09-25

**Authors:** Junhu Su, Achyut Aryal, Zhibiao Nan, Weihong Ji

**Affiliations:** 1 College of Grassland Science, Key Laboratory of Grassland Ecosystem (Ministry of Education), Pratacultural Engineering Laboratory of Gansu Province, Sino-U. S. Centers for Grazing land Ecosystem Sustainability, Gansu Agricultural University, Lanzhou, 730070, P. R. China; 2 Gansu Agricultural University-Massey University Research Centre for Grassland Biodiversity, Gansu Agricultural University, Lanzhou, 730070, P. R. China; 3 State Key Laboratory of Grassland Agro-ecosystems, College of Pastoral Agriculture Science and Technology, Lanzhou University, Lanzhou, 730000, P. R. China; 4 Institute of Natural and Mathematical Sciences, Massey University, Private Bag 102 904 North Shore Mail Centre 0632, Auckland, New Zealand; Sichuan University, CHINA

## Abstract

Disturbances, both human-induced and natural, may re-shape ecosystems by influencing their composition, structure, and functional processes. Plateau zokor (*Eospalax baileyi*) is a typical subterranean rodent endemic to Qinghai-Tibetan Plateau (QTP), which are considered ecosystem engineers influencing the alpine ecosystem function. It is also regarded as a pest aggravating the degradation of overgrazed grassland and subject to regular control in QTP since 1950s. Climate change has been predicted in this region but little research exists exploring its impact on such subterranean rodent populations. Using plateau zokor as a model, through maximum entropy niche-based modeling (Maxent) and sustainable habitat models, we investigate zokor habitat dynamics driven by the future climate scenarios. Our models project that zokor suitable habitat will increase by 6.25% in 2050 in QTP. The predication indicated more threats in terms of grassland degradation as zokor suitable habitat will increase in 2050. Distribution of zokors will shift much more in their southern range with lower elevation compare to northern range with higher elevation. The estimated distance of shift ranges from 1 km to 94 km from current distribution. Grassland management should take into account such predictions in order to design mitigation measures to prevent further grassland degradation in QTP under climate change scenarios.

## Introduction

Disturbances, both human-induced and natural, may reshape ecosystems by influencing their composition, structure, and functional processes [1, 2, 3]. Climate change, one of the key natural disturbances affecting populations of organisms, has long been considered the greatest challenge to natural and biodiversity conservation [4, 5, 6]. It is widely believed that both historical and contemporary climate change affect populations of organisms. Historical climate change have contributed to the evolutionary process of organisms and even triggered the speciation process [7, 8]. Current and future climate changes are predicted to potentially affect population size, survival and distribution of organisms [4, 9, 10]. Climate has long been considered critical in altering population dynamics through altering the factors such as population growth rate, migration and overwintering [11, 12]. Distributions of mountain biota are predicted to move toward poles or higher altitude as a result of global climate warming [1, 13]. Indeed, many insect species have shifted their ranges to higher latitudes and altitudes during recent climate warming [14].

However, existing studies that investigate the effect of climate change mainly focus on endangered or invasive species [15, 16, 17]. Studies on non-threatened native species are scarce. In areas where the natural ecosystems suffer from degradation due to anthropomorphic disturbances, some native animals are known to become problematic and aggravate the degradation [18]. With the ongoing disturbances from climate changes, many native species potentially become problematic causing further challenges for management of ecosystems and biodiversity [19]. Understanding the effects of climate change on these species is essential for current and future species management and biodiversity conservation under the ongoing climate change scenarios.

Climate change can potentially affect both aboveground and subterranean biota. It is reported that the distribution of subterranean species have been affected by historical climatic factors [20]. But the knowledge on the influence of current and future climate changes on subterranean species is scarce [21].

The Qinghai-Tibetan Plateau (QTP), the largest plateau in the world, occupying 2.5 million km^2^ with an average altitude of 4000 m above sea level, is a region sensitive to climate changes. QTP is also one of the largest rangeland areas in the world. Like many alpine grassland in world, large areas of rangeland in QTP suffer various degree of grassland degradation [22]. On QTP, large areas are designated as protected conservation area, in which hunting endangered species are prohibited. However, except small proportion of these areas that have been totally protected (core areas), large proportion of these areas are still inhabited by local farming communities where alpine grassland are used for farming (yak, sheep and horse etc) [22, 23]. Rangeland in these areas are degraded and overgrazing is considered the main factor causing grassland degradation, which is aggravated by activities of small mammals such as pika [24] and subterranean rodents such as zokors [23].

Alpine zokors of subfamily Mysopalacinae include two genera, *Eospalax* and *Myospalax*. They are typical subterranean rodents that inhabit alpine prairie, meadow and farmland [25, 26, 27]. When present at natural densities, they play important role in the ecosystem function, such as nutrient cycling, soil structure and vegetation composition, and are regarded as ecosystem engineers [28]. However, in some degraded rangeland, they are known to form dense populations, reducing crop production, competing with livestock, causing soil erosion and contributing to further rangeland degradation [23]. Large scale culling of zokors using toxins has been the main method for controlling zokor populations and such control are often conducted in sensitive and fragile ecosystems such as steppe and semi-desert grasslands. Significant population reduction or eradication of these species has had significant negative impacts on biodiversity and potentially on other ecosystem aspects and function such as soil structure, nutrient cycling, tropic interactions and plant community structure [25].

Despite the control, this species continue to challenge rangeland management due to their roles in grassland ecosystem health. Climate change is known to have affected distribution and life history characteristics of many species, especially species that inhabit alpine regions [29]. The potential effect of climate change on zokor populations has not been examined. Such study is important for effectively planning the future management and preventing further degradation of alpine grassland ecosystem.

The distribution and population density of zokors are limited by elevation, precipitation, vegetation and anthropogenic disturbance. It is generally agreed that climatic changes influence species’ distributions through species-specific physiological thresholds of temperature and precipitation tolerance [4]. With climate change, some habitats that are not currently suitable for zokors may be suitable in the future based on the direction of change in habitat conditions. Global average temperatures have increased by 0.2°C per decade since the 1970s, and global average precipitation increased by 2% in the last 100 years [30]. On the QTP, the climate change is mainly reflected by milder winters [22], which has resulted in phenological changes such as earlier breeding or peak in biomass of plants [4]. Observed impacts of climate change have been reported in relation to grassland ecosystems, its productivity, plant community composition and distribution, and soils [31]. Therefore climate change can potentially alter the temporal and spatial patterns of food availability and soil tunneling condition for zokors and cause the shift in distribution of these species. In this study, we use the plateau zokor (*E*. *baileyi*) as the focal species. Through the maximum entropy niche-based (Maxent) modeling and sustainable habitat modeling we analyse the potential effect of predicted climate change on the distributions of alpine subterranean rodents. Our objectives are to: (1) determine the environmental parameters that influence current plateau zokor distribution at QTP; (2) predict future changes in plateau zokor distribution and range shifts and (3) provide baseline information for management of alpine rangeland under climate change scenarios.

## Materials and Methods

### Study area and presence data collection

**Fig 1:**
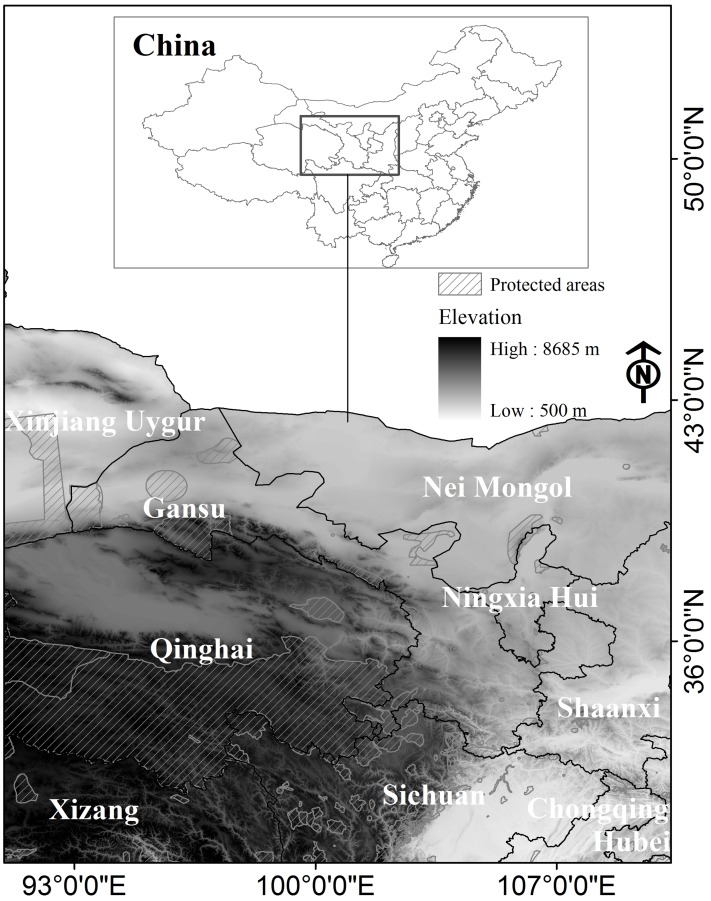
Study areas with elevation (https://lta.cr.usgs.gov/GMTED2010) and protected areas networks [32].

Our study areas covered the Qinghai-Tibetan Plateau (QTP) (Fig 1). We used world protected area network data from IUCN and UNE-WCMC 2015 [32] for this study. We collected the GPS location data of the plateau zokor species from published papers [33, 34], unpublished reports, and field surveys over 5 years from 2008 to 2012 (Fig 1). We identified 194 plateau zokor locations. We used 1-km^2^ grid cells for the maximum entropy niche-based (Maxent) modeling and sustainable habitat modeling [35]. We use one plateau zokor presence per cell and removed the repeated location data. A final 99 GPS points of plateau zokor presence are included for further analysis.

### Data Modeling

We used Maxent model, a widely use tool for Species Distribution Models (SDMs), to predict current and future suitable habitat of zokor [35]. SDMs have been usedto understand different ecological questions relate to habitat preference and suitability, distribution, future prediction, conservation issue and evolutionary study of species [35]. Here, we used Maxent model to understand zokor distribution in different time series. We used current presence points of zokor and extracted nineteen bioclimatic variables of study area from www.worldclim.org [36], land use and land cover [37] (http://www.glcn.org). In ArcGIS, we prepared aspect and slope layers using a digital elevation model (DEM) (https://lta.cr.usgs.gov/GMTED2010) layer and clipped all variables to our study areas. We then extracted the values of each variable corresponding to the occurrence locations of plateau zokor to perform correlation analysis. After removing 11 highly correlated variables (>0.85; S1 Fig), we used the remaining 12 variables for our final analysis.

The MIROC5 (Model for Interdisciplinary Research on Climate) and General Circulation Model (GCM) [38] were used to project the future climate [39, 40]. GCM data were downscaled using the delta method and bias corrected by world clim’s current climate (http://worldclim.org/). We ran the MIROC5 model using the Representative Concentration Pathway 4.5, a “middle-of the-road” GHG (Green House Gas) scenario. We used two time periods for analysis: current and 2050 (average for 2041–2060; http://worldclim.org/cmip5_30s).

## Results

### Accuracy of species distribution models

**Fig 2:**
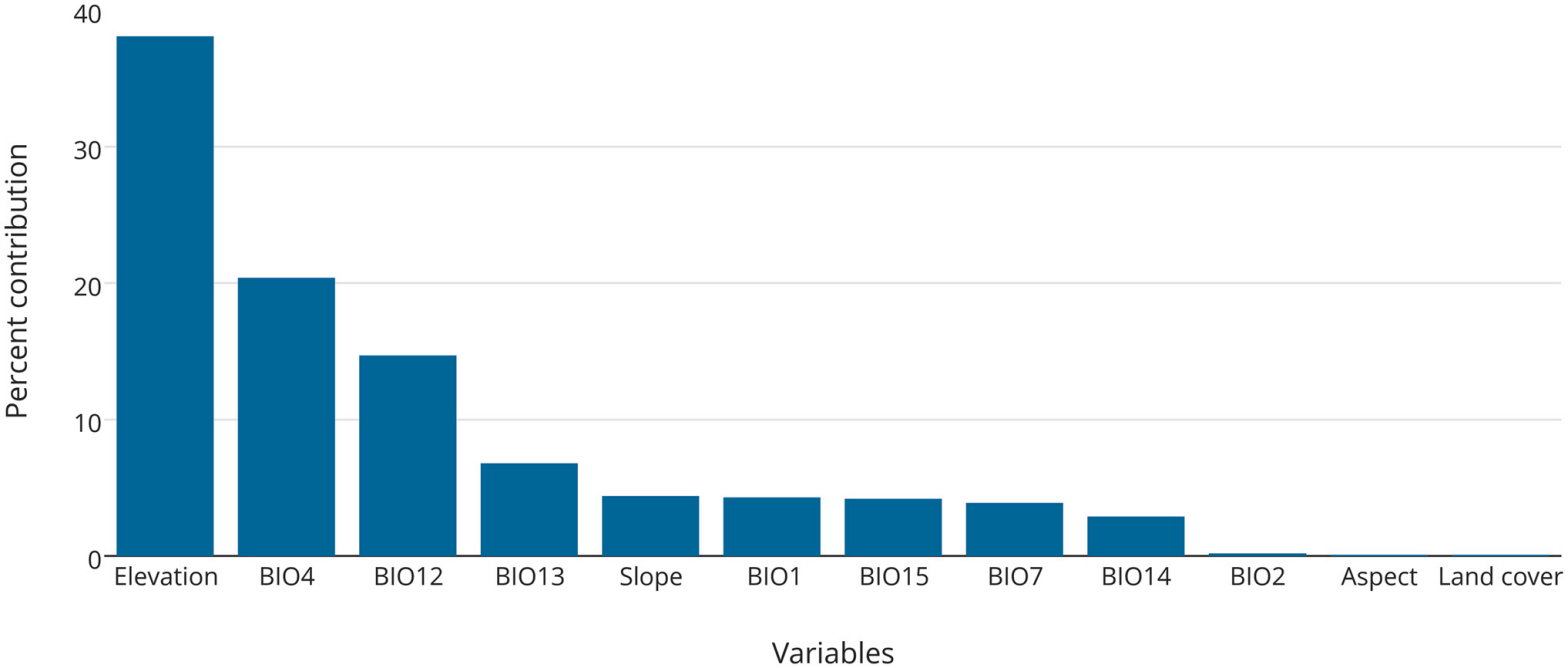
Contribution variables for modeling.

Overall, elevation (38%), precipitation of the driest month (BIO14; 20.4%) and annual precipitation (BIO12; 14.7%) were main contributing factors which contributed 72% for identification of zokor habitats (Fig 2). Habitat suitability of zokor is more related to precipitation related variables with an overall contribution of 50%, followed by temperature (BIO1; 4.3%), slope, aspect, land cover (Fig 2). The jackknife test also showed that elevation and precipitation were the main important variables (S1 Fig). The habitat suitability model was the best model as it has the omission rate very close to the predicted omission (cumulative threshold) (S2 Fig). This model described plateau zokor habitat very well (AUC = 0.99; S3 Fig). Response curves of each environmental variable have affected the distribution and predication of plateau zokor habitat and showed their effect on future prediction and current habitat of suitability of plateau zokor (S4 Fig).

### Effect of precipitation, rainfall and other variables on plateau zokor habitat

**Fig 3:**
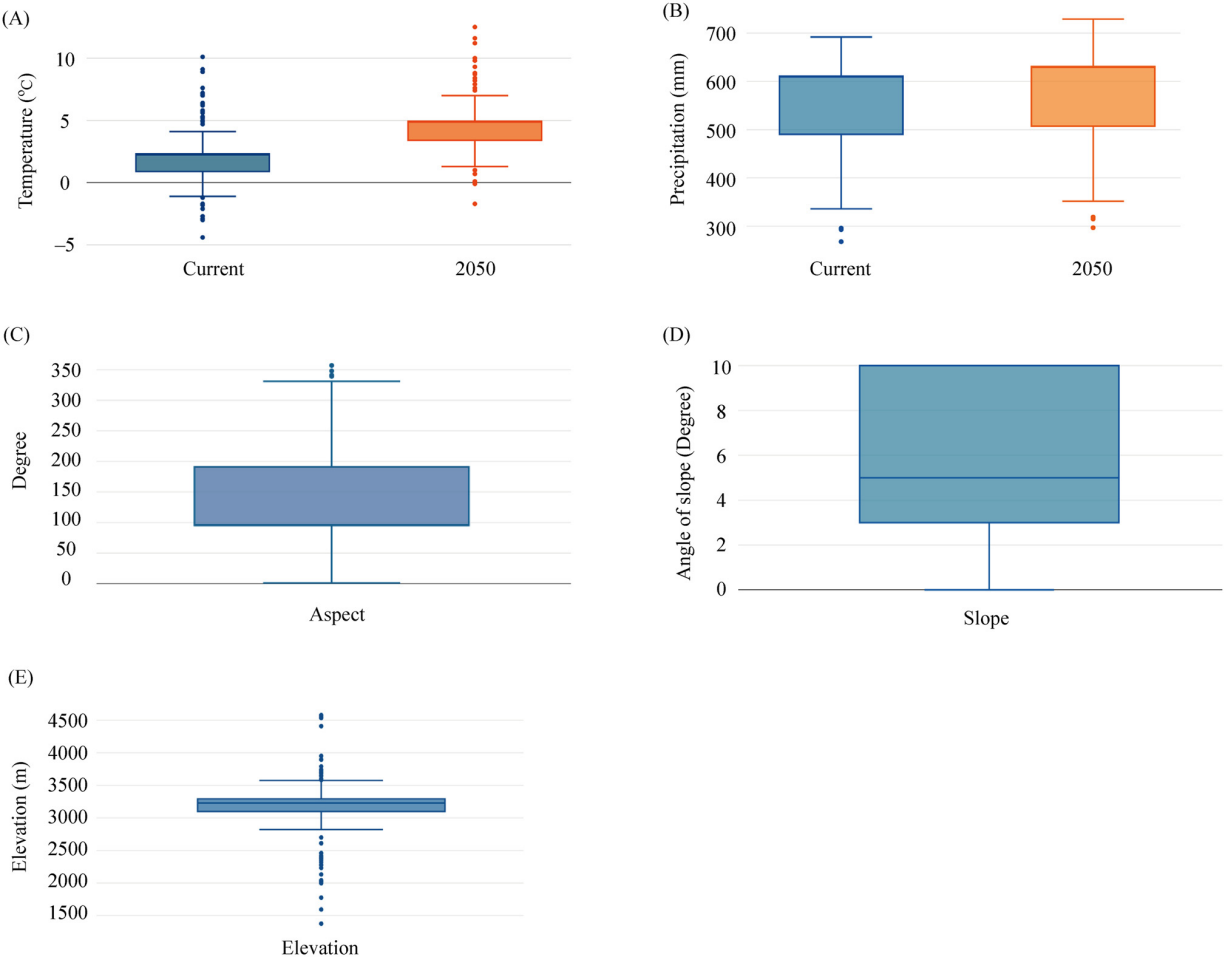
Current and future temperature (a), precipitation (b) and current suitable habitat aspect (c), slope (d) and elevation (e), in plateau zokor habitat.

The mean annual temperatures of current suitable habitat of plateau zokor ranges from -4.4°C to 10.1°C (Fig 3A). The mean annual precipitations of current plateau zokor habitat ranges from 268 mm to 692 mm (Fig 3B). Based on the predicted climate change, the mean annual temperature will increase by 2.4°C in 2015 (current: 2.1+2.1°C; 2050: 4.6+2.1°C; means + standard deviation, SD) in plateau zokor habitat (Fig 3A) and mean precipitation will increase by 19 mm (current: 558.63+95.53; 2050: 577.12+98.60; mean+SD) (Fig 3B). The elevation was the main factors for predicting plateau zokor distribution. The mean altitude of areas that suitable as plateau zokor habitat was 3186 m±510; SD, mean aspect 140 (south east) and mean degree of slope 5.8 (Fig 3C and 3E).

### Predicting current and future habitat for plateau zokor

The total areas of current suitable habitat for plateau zokor species is 270074 km^2^ in China. Our model showed that currently Qinghai province provides the largest area of suitable habitat of plateau zokor (51.0%; 138223 km^2^) followed by Gansu province (35.9%; 96996 km^2^), Sinchuan province (7.6%; 20528 km^2^) and Xizang province (1.2%; 3428 km^2^) (Table 1).

**Table 1:** Current and future suitable zokor habitat in different province of China.

Province	Current suitable habitat in km2	Future (2050) suitable habitat in km2	Change in %	Current suitable habitat in %
Gansu	96996.35	101273.31	+4.22	35.91
Ningxia	9661.61	10501.90	+8.00	3.58
Qinghai	138223.00	149243.64	+7.38	51.18
Shaanxi	1235.43	387.41	-218.89	0.46
Sichuan	20528.76	23066.24	+11.00	7.60
Xizang	3428.19	3561.90	+3.75	1.27
Total	270073.39	288034.45	+6.24%	
Suitable habitat within protected area system	90452.74	99116.15	+8.74%	

**Fig 4:**
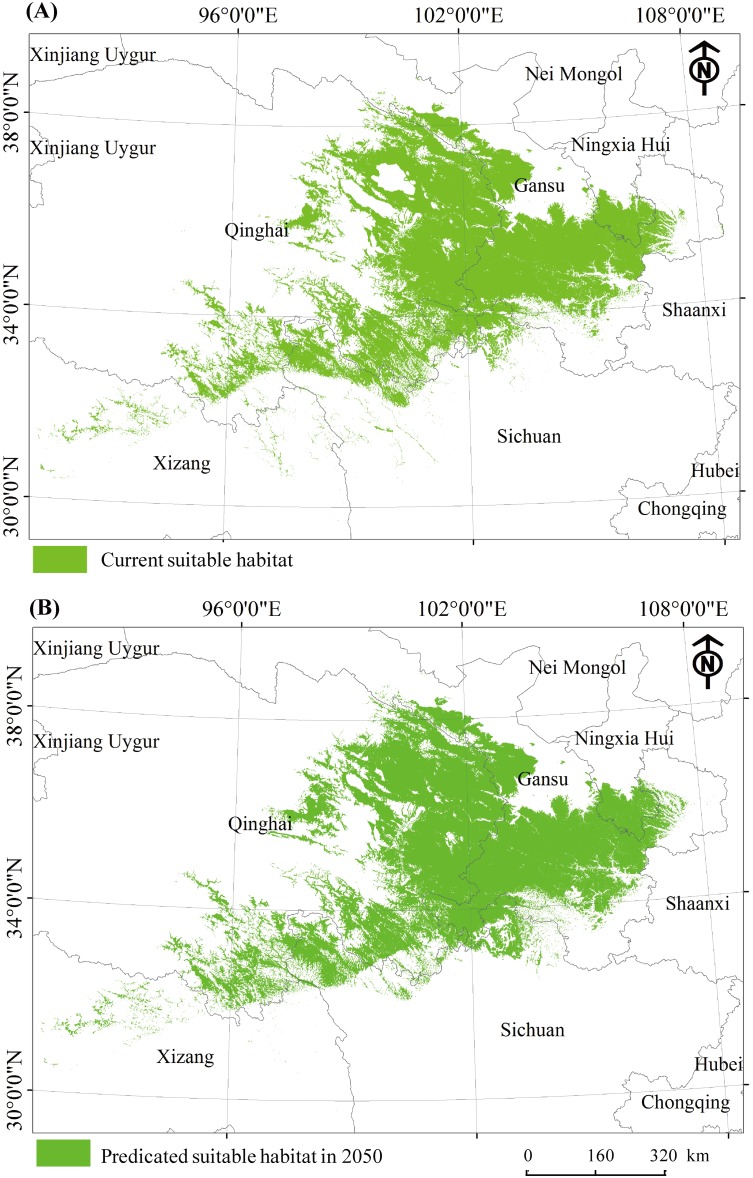
The current (a) and predicated suitable habitat (b) of plateau zokor.

Our model predicated that the plateau zokor suitable habitats will increase by 6.2% (i.e. 288080 km^2^) according to the scenario of changing in global temperature and precipitation (Fig 4; Table 1). The highest increase in future suitable habitat predicted (7.0%; 138223 km^2^ to 149243 km^2^) is in Qinghai province, followed by Gansu province (4.0%; 96996 km^2^ to 101273 km^2^) and Sichuan province (11.0%; 20528 km^2^ to 23066 km^2^) (Table 1). Currently 33.0% (90452 km^2^) of total plateau zokor suitable areas lied inside the protected areas. The predicted climate change will results in an 8.7% increase in plateau zokor suitable areas inside the protected areas. The plateau zokor suitable habitat is predicted to reach 99116 km^2^ inside protected areas by 2050 (Fig 4).

### Range shifting

Our result showed that a higher degree of shift of plateau zokor suitable habitat will occur at lower elevation compare to higher elevation (Fig 4). In the southern range of current suitable habitat, plateau zokor is predicted to move further south by 4 km to 70 km distance, however their potential range shift in northern range would be less, ranging from 1 km to 14 km (Fig 4). Most of plateau zokor habitat is predicted to expand and shift towards north-east of current suitable habitat (Fig 4).

## Discussion

### Factors that influence current zokor distribution

Our best model, the habitat suitability model, was very close to the predicted omission rate, which also described plateau zokor habitat very well. Response curves of each environmental variable have affected the distribution and predication of plateau zokor habitat which showed their effect on the predicted future and current habitat suitability of plateau zokor.

Our SDM model, which incorporates 15 variables, predicted that elevation and precipitation are the most important variables that influence current plateau zokor distribution on QTP. Precipitation related variables, annual precipitation, precipitation of driest month and slope, aspect, land cover make up 50% of overall contributions to habitat suitability.

The elevation and precipitation are the most important factors affecting soil moisture and plateau zokors’ energetic balance. The plateau zokor burrow systems maintain relatively high moisture content, 79.0–87.3% [41, 42] when aboveground humidity was 31.7–53.3%. The high humidity contributes to economizing water balance by zokors [43]. Zeng *et al* (1984) found that the water loss of plateau zokor through evaporation, (0.79 mg/g) is lower than that of opening burrow animals such as plateau pika (*Ochotona curzoniae*) (1.38 mg/g) [41]. In addition, precipitation is also an important factor affecting energetic cost of tunneling by subterranean animals. High precipitation increase soil moisture and decrease soil compaction, which reduce the energetic cost of tunneling, making areas that were unsuitable for tunneling become suitable habitat for subterranean animals. In contrast, for tunneling small mammals such as plateau pikas (*O*. *curzoniae*) that are active above ground, temperature, instead of precipitation is the most important factor predicts habitat suitability [44, 45].

### Range shifts of plateau zokor under the climate change scenarios

Niche model that correlate known occurrences of species with environmental variables and predict their potential distributions is a good method for assessing the risk of pest establishment [46]. Using this method, we predicted a range expansion of plateau zokor by 1km to 96 km from the current distribution in QTP. Interestingly, we found different patterns of range shift of zokor populations in high altitude and low altitude. In lower range of current suitable habitat, plateau zokor distribution is predicted to shift toward further lower (south) area by 4 km to 70 km. Less degree of range shift is predicted in northern region, by 1km to 14 km towards north (Fig 4). Plateau zokor is the only zokor species inhabit areas above 3000m. Their predicted range shift is likely to realize. At lower altitude, below 3000m, two zokor species, plateau zokor and Gansu zokor coexist. The potential interaction between the two species is likely to happen. Therefore, under climate change scenarios, there are three possible outcomes of their distribution patterns: 1) Increased overlap in distribution between the two species, 2) Gansu zokor would outcompete Plateau zokor resulting in restricting the expansion by plateau zokor at lower altitude or 3) Plateau zokor would out compete Gansu zokor resulting in expansion of plateau zokor in areas of lower altitude and reduction in distribution of Gansu zokor. Range expansion to lower altitude present plateau zokor with a new type of habitat, agricultural land. Abiotic factors, biotic interactions and dispersal factors are three key elements that determine distribution of species and their abundances at different scales [47]. How plateau zokor would respond to this new habitat worth investigation. SDMs are based on a niche conservatism hypothesis and particularly used to project the locations species will occupy following climate change [48, 49]. Niche is evolutionary, but in the evolution of the short history, it’s conservative [46, 50]. Variations in life history and foraging ecology can serve as a basis for grouping species’ responses to climate change [51]. Niche conservatism, adaptation and plasticity play important roles in response to climate change [52]. Current trend of climate change on QTP predicts phenological changes such as earlier thaw of soil and plant germination. This may lead to changes of zokor foraging activities and life history traits such as breeding patterns, and this also worth to investigate.

#### Implications for rangeland management under climate change

Rangeland management should anticipate how disturbance such as climate change may alter the geographic distributions of species that potentially contribute to the rangeland degradation. In areas that new invasion of zokors is predicted, the negative impact of this species on grassland health is also likely. Overgrazing is considered the main factor causing grassland degradation [23, 24], a condition at which the zokors are known to be problematic [53]. Therefore it is important to determine optimal grazing level of alpine grassland so that it can be effectively managed to maintain biodiversity and grassland health, in order to build the resilience against disturbances by such new invaders. Further empirical studies on how zokors and associated native species respond to climate change are important for informing future management and conservation of native alpine ecosystems. Conservation planning should incorporate not only the effects of climate change on the aboveground livings but also on the subterranean biota.

## Supporting Information

S1 Fig The following picture shows the results of the jackknife test of variable importance. (TIF)

S2 Fig The picture shows the omission rate and predicted area as a function of the cumulative threshold. (TIF)

S3 Fig Receiver operating characteristic (ROC) curve for the variables used for modeling. (TIF)

S4 Fig Response curves. (TIF)

S1 Table Variables use for modeling. (XLSX)

S2 Table Cumulative and logistic thresholds and corresponding omission rates used for modeling. (DOCX)
