# Supplementary figures and images for: Climate Change-Induced Range Expansion of a Subterranean Rodent: Implications for Rangeland Management in Qinghai-Tibetan Plateau
Source: PLoS One. 2015 Sep 25;10(9):e0138969. doi: 10.1371/journal.pone.0138969 (PMC4583544; doi:10.1371/journal.pone.0138969)

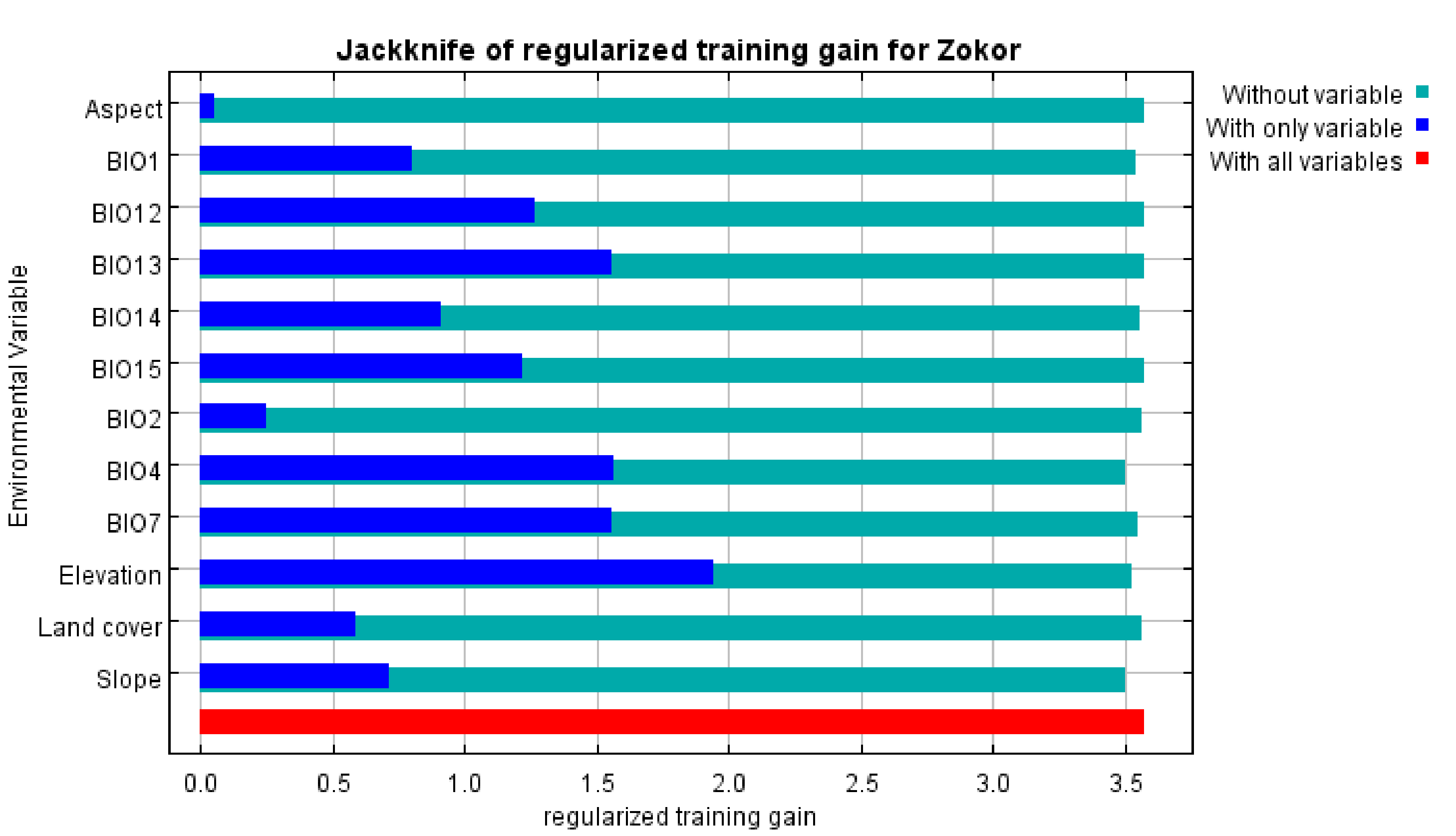

Supplement: S1 Fig — (TIF) [file pone.0138969.s001.tif]

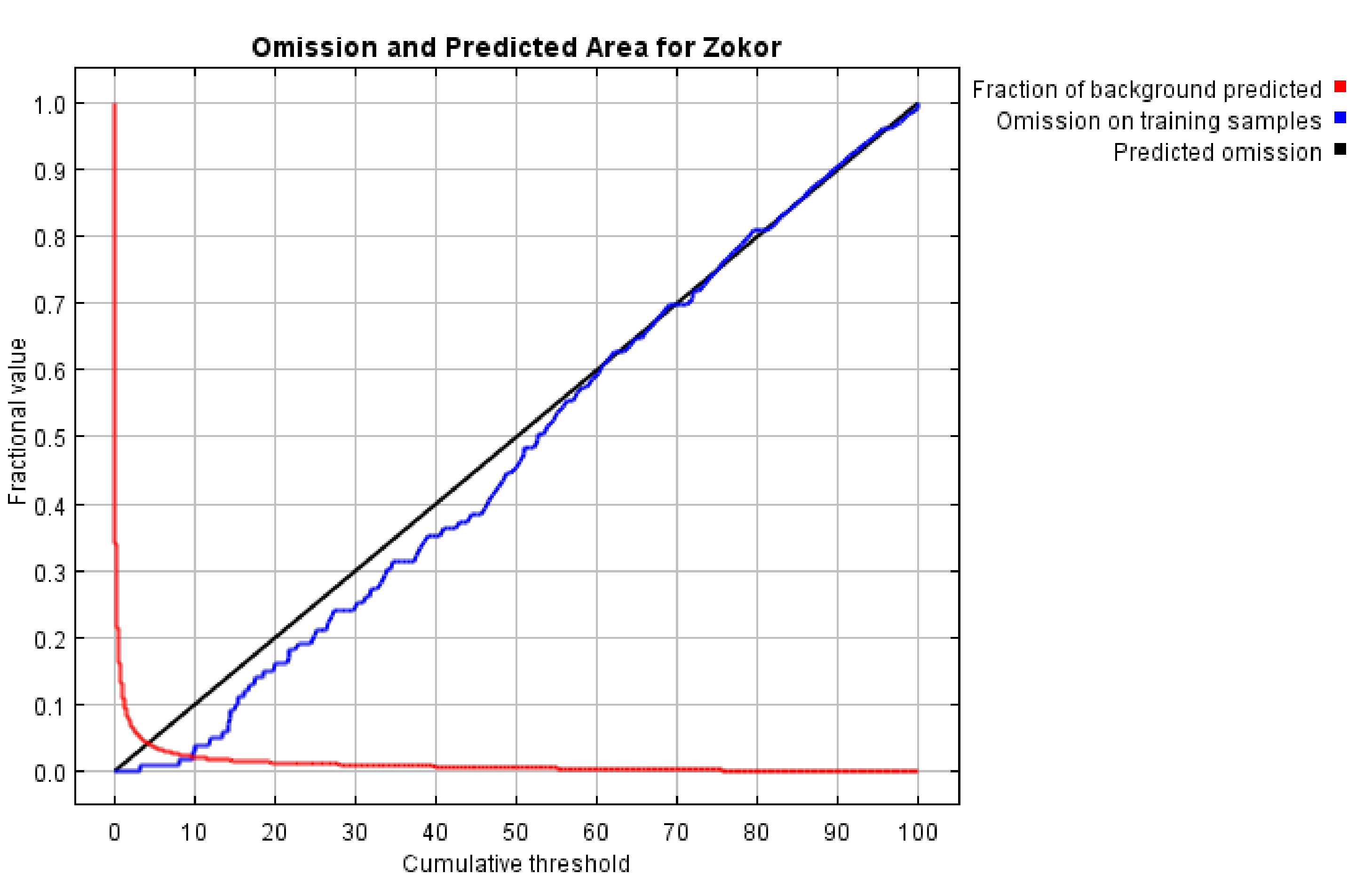

Supplement: S2 Fig — (TIF) [file pone.0138969.s002.tif]

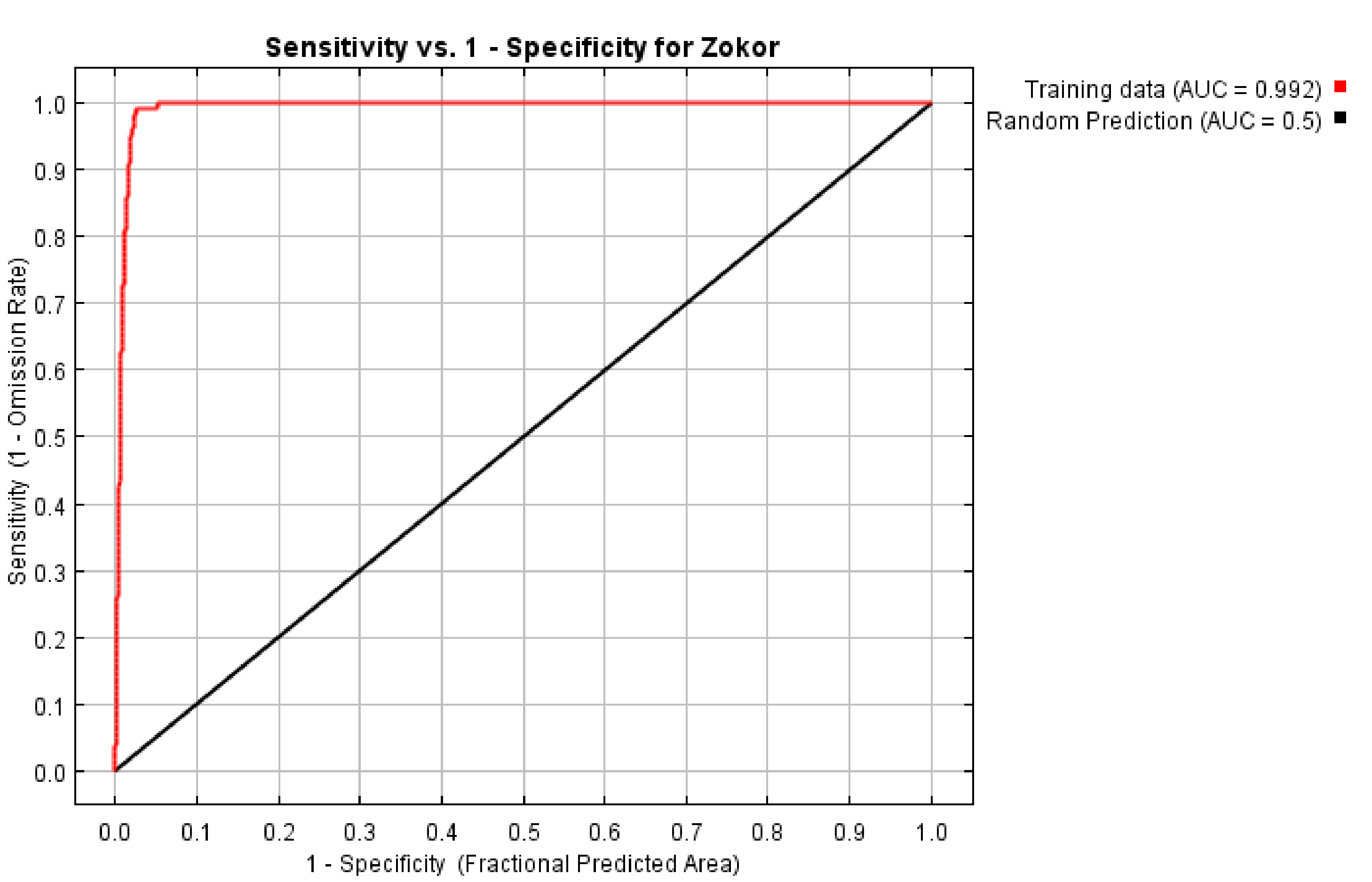

Supplement: S3 Fig — (TIF) [file pone.0138969.s003.tif]

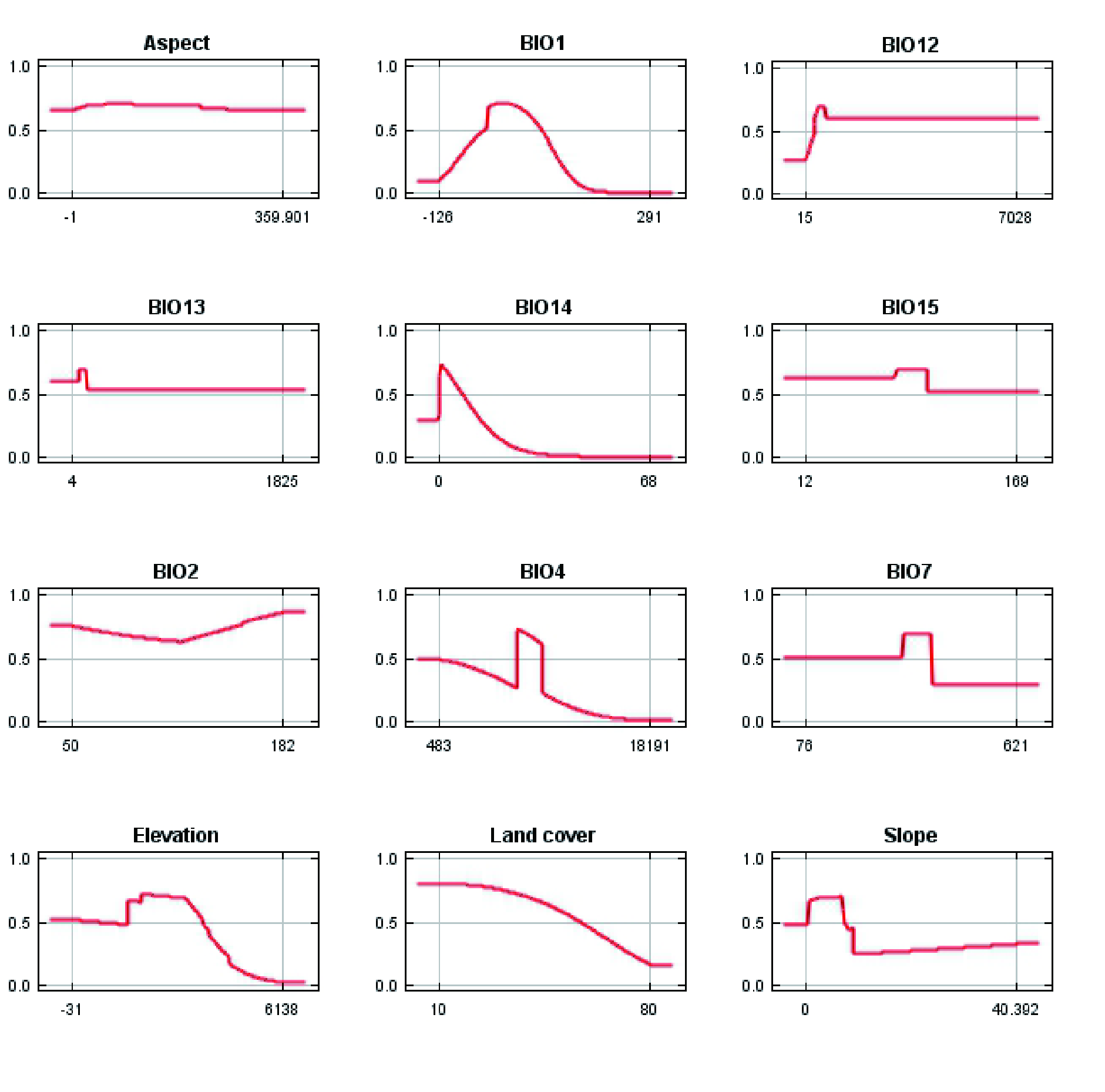

Supplement: S4 Fig — (TIF) [file pone.0138969.s004.tif]
