# Supplementary material for: Climate Change-Induced Range Expansion of a Subterranean Rodent: Implications for Rangeland Management in Qinghai-Tibetan Plateau
Source: PLoS One. 2015 Sep 25;10(9):e0138969. doi: 10.1371/journal.pone.0138969 (PMC4583544; doi:10.1371/journal.pone.0138969)
Supplement: S2 Table — (DOCX) [file pone.0138969.s006.docx]

## Table 2: Variables use for modelling

| ***SN*** | ***Variables*** | ***Sources*** |
| --- | --- | --- |
| 1 | BIO1(Annual Mean Temperature) | http://www.worldclim.org/bioclim (Hijmans et al. 2005) |
| 2 | BIO2 (Mean Diurnal Range) |  |
| 3 | BIO3 (Isothermality) |  |
| 4 | BIO4(Temperature Seasonality) |  |
| 5 | BIO5 (Max Temperature of Warmest Month) |  |
| 6 | BIO6 (Min Temperature of Coldest Month) |  |
| 7 | BIO7(Temperature Annual Range) |  |
| 8 | BIO8 (Mean Temperature of Wettest Quarter) |  |
| 9 | BIO9 (Mean Temperature of Driest Quarter) |  |
| 10 | BIO10 (Mean Temperature of Warmest ) |  |
| 11 | BIO11(Mean Temperature of Coldest Quarter) |  |
| 12 | BIO12(Annual Precipitation) |  |
| 13 | BIO13(Precipitation of Wettest Month) |  |
| 14 | BIO14(Precipitation of Driest Month) |  |
| 15 | BIO15(Precipitation Seasonality (Coefficient of Variation) |  |
| 16 | BIO16 (Precipitation of Wettest Quarter) |  |
| 17 | BIO17(Precipitation of Driest Quarter) |  |
| 18 | BIO18(Precipitation of Warmest Quarter) |  |
| 19 | BIO19(Precipitation of Coldest Quarter) |  |
| 20 | Land cover | <http://www.glcn.org> (Latham et al. 2014) |
| 21 | Aspect | <https://lta.cr.usgs.gov/GMTED2010> (Danielson and Gesch 2011) |
| 22 | Slope |  |
| 23 | Elevation |  |

**Table 3**. cumulative and logistic thresholds; and corresponding omission rates used for modelling.

| Cumulative threshold | Logistic threshold | Description | Fractional predicted area | Training omission rate |
| --- | --- | --- | --- | --- |
| 1.000 | 0.003 | Fixed cumulative value 1 | 0.118 | 0.000 |
| 5.000 | 0.046 | Fixed cumulative value 5 | 0.037 | 0.010 |
| 10.000 | 0.153 | Fixed cumulative value 10 | 0.022 | 0.040 |
| 3.254 | 0.026 | Minimum training presence | 0.051 | 0.000 |
| 14.890 | 0.271 | 10 percentile training presence | 0.017 | 0.091 |
| 9.637 | 0.143 | Equal training sensitivity and specificity | 0.023 | 0.020 |
| 7.998 | 0.102 | Maximum training sensitivity plus specificity | 0.026 | 0.010 |
| 2.031 | 0.011 | Balance training omission, predicted area and threshold value | 0.071 | 0.000 |
| 7.261 | 0.089 | Equate entropy of thresholded and original distributions | 0.028 | 0.010 |

**Table 4:** Current and future suitable zokor habitat in different province of China

| **Province** | Current suitable habitat in km^2^ | Future (2050) suitable habitat in km^2^ | Change in % | Current suitable habitat in % |
| --- | --- | --- | --- | --- |
| Gansu | 96996.35 | 101273.31 | +4.22 | 35.91 |
| Ningxia Hui | 9661.61 | 10501.90 | +8.00 | 3.58 |
| Qinghai | 138223.00 | 149243.64 | +7.38 | 51.18 |
| Shaanxi | 1235.43 | 387.41 | -218.89 | 0.46 |
| Shanxi | 0.97 | 16.05 | +93.93 | 0.00 |
| Sichuan | 20528.76 | 23066.24 | +11.00 | 7.60 |
| Xizang | 3428.19 | 3561.90 | +3.75 | 1.27 |
| Nei Mongol | 0.54 | 0 | 0 |  |
| **Total** | **270074.9** | **288050.5** | **+6.24%** |  |
| **Suitable habitat within protected area system** | **90452.74** | **99116.15** | **+8.74%** |  |
